# Barriers and facilitators to sexual and reproductive health services among adolescents and young adults in Sidama region, Ethiopia: a qualitative study

**DOI:** 10.1186/s12978-026-02400-2

**Published:** 2026-06-24

**Authors:** Melaku Birhanu Alemu, Jaya AR Dantas, Zohra S Lassi, Richard Norman, Gavin Pereira, Gizachew A Tessema

**Affiliations:** 1 Curtin School of Population Health, Faculty of Health Sciences, Curtin University, Perth, Australia; 2 Population Health Sciences Institute, Newcastle University, Newcastle Upon Tyne, UK; 3 Department of Health Systems and Policy, Institute of Public Health, University of Gondar, Gondar, Ethiopia; 4 School of Population Health, College of Health, Adelaide University, Adelaide, Australia; 5 WHO Collaborating Centre for Climate Change and Health Impact Assessment, Perth, Australia; 6 enAble Institute, Faculty of Health Sciences, Curtin University, Perth, Australia; 7 Faculty of Medicine, Universitas Negeri Malang, Malang, Indonesia; 8 Institute for Health Research, University of Notre Dame, Fremantle, Australia

**Keywords:** Sexual health, Reproductive health, SRH, Adolescents, Young adults, Preference, Young people, Utilisation, Ethiopia

## Abstract

**Background:**

Adolescents and young adults (AYAs) in Ethiopia face multiple challenges in accessing sexual and reproductive health (SRH) services. Understanding the barriers and facilitators to SRH service uptake is essential for designing tailored interventions. Therefore, this study aimed to explore barriers and facilitators to SRH service uptake from the perspectives of AYAs and stakeholders in southern Ethiopia.

**Methods:**

We conducted a qualitative study in the Sidama region, Southern Ethiopia using focus group discussions (FGDs) with AYAs recruited from schools, communities, and a non-governmental organisation. A supplemental FGD was conducted with government officials, clinicians and SRH experts to capture stakeholder perspectives. The FGD was conducted in the national language (*Amharic*), which was then transcribed and translated into English for analysis. A thematic content analysis was used to present emerging themes in the FGDs. A ranking of barriers and facilitators was conducted to identify the most influential factors for SRH service uptake. NVivo 15 was used for data management and organisation for the thematic analysis.

**Results:**

Nine FGDs were conducted with 56 AYAs and seven experts, with each FGD comprising 6–8 participants. We identified four major themes: 1) health system and policy environment, 2) service access and delivery, 3) user experience and outcomes, and 4) sociocultural and economic determinants. Participants ranked service quality, cost, and privacy higher in their influence on determining SRH service uptake. Stigma, religious beliefs, poverty, and urban migration were also identified as barriers to SRH service access among AYAs.

**Conclusions:**

SRH service uptake among AYAs was shaped by their interconnected health system and policy environment; service access and delivery; user experiences and outcomes, and sociocultural and economic determinants. Barriers and facilitators from service access and delivery theme such as service quality, privacy and health system factors such as cost emerged as the most influential drivers shaping service uptake. Policy and programme efforts should consider improving service quality, strengthening confidentiality, and reducing financial barriers. The findings may also inform future quantitative research examining AYAs’ preferences for SRH service.

**Supplementary Information:**

The online version contains supplementary material available at https://doi.org/10.1186/s12978-026-02400-2.

## Abstract (plain language summary)

Young people in Ethiopia often face challenges when accessing sexual and reproductive health (SRH) services. These challenges may include poor service quality, lack of privacy, high costs, and cultural and economic barriers. This study examined at what helps or prevents adolescents and young adults (AYAs) from using SRH services and explored which service features matter most to them.

We conducted focus group discussions (FGDs) in the Sidama region with 56 young people from schools, communities, and a charitable organisation working with children and young people. We also conducted an FGD with seven stakeholders, including government officials and healthcare providers, to explore their views. The FGDs were conducted in Amharic, and audio recorded. The recordings were transcribed and translated into English. Common ideas and patterns were then organised into themes.

We identified four major themes that are important to young people’s uptake of SRH services: 1) health system and policy environment, 2) service access and delivery, 3) user experience and outcomes, and 4) sociocultural and economic determinants. The most important factors influencing whether young people used SRH services were service quality, privacy, and cost. Stigma, religious beliefs, poverty, and urban migration further hindered access to SRH services.

We recommend that efforts to improve SRH service use among AYAs consider service quality, privacy, and affordability, alongside broader social and economic barriers. These findings may inform future research and support policy and programme design to better align SRH services with the needs and preferences of AYAs in Ethiopia.

**Supplementary Information:**

The online version contains supplementary material available at https://doi.org/10.1186/s12978-026-02400-2.

## Introduction

Adolescents and young adults (AYAs) are at a crucial stage of development as they transition from childhood to adulthood [1]. This group includes individuals aged 10 to 24 years and represents approximately 24% of the global population. Of this population, 21% live in sub-Saharan African countries [2]. In 2024, it was estimated that AYAs constitute approximately one-third of the African population. In the same year, more than 37 million AYAs were living in Ethiopia [2].

In low- and middle-income countries (LMICs), nearly one in three young adults aged 20 to 24 had given birth during adolescence (10 to 19 years), where half of these adolescent mothers were aged 17 years or younger [3]. In Ethiopia, the burden of sexual and reproductive health (SRH) issues among AYAs remains high. For example, a study conducted in 2019 showed that approximately one in three pregnancies among currently pregnant AYAs in western Oromia was unintended [4]. A study conducted across 13 major urban towns in Ethiopia found that 14% of participants aged 18 to 29 years had experienced at least one unwanted pregnancy in 2022 [5]. In regard to abortion, adolescents have the highest abortion rate among all age groups when adjusted for sexual activity [6]. Although the prevalence of HIV among AYAs was relatively low [7], nearly one in three young people aged 18 to 29 years engaged in high-risk sexual behaviours. Around 14% of students from high school and university students in Ethiopia had sexually transmitted infections (STIs), with the highest prevalence being university students (14.5%) [8].

Previous studies in Ethiopia have consistently shown a low level of SRH service utilisation across regions [9–11]. In 2019, only 54% of married women aged 15 to 24 years used modern contraceptives [12]. Adolescent women aged between 15 to 19 years were more likely to use legal abortion services than clandestine abortion options compared to than other age groups in 2014 [6]. The early treatment of STIs among individuals attending youth-friendly services in Bahir Dar was just 28% in 2023 [13]. In a school-based study, the utilisation of SRH services was reported to be 44% in southern Ethiopia in 2019 [14]. This evidence highlighted persistent challenges in improving SRH service access and uptake among AYAs.

Several factors influence AYAs' access to SRH services in Ethiopia. These include age, knowledge, stigma, parent–child communication and family support, access to social media, school enrolment, sexual initiation and previous STI diagnosis, service availability and quality concerns, peer pressure, geographic location, economic status, and education [13–18]. In 2023, AYAs demonstrated preferences for service providers, the channels through which they access services, and the timing of service provision, all of which are important considerations for improving SRH services [19].

To address these challenges, the World Health Organization (WHO) launched the Global Accelerated Action for the Health of Adolescents (AA-HA!) programme in 2017 to guide the implementation of health interventions targeting AYAs [20]. In addition, WHO developed global standards for quality healthcare services for adolescents to support national health interventions [21]. Aligned with these global efforts, the Ethiopian government introduced a national adolescent and youth health strategy in 2016 to improve service utilisation among AYAs, which was revised in 2021 to cover the period 2021 to 2025 [22].

While some studies have investigated adolescents’ preferred service environments s in parts of southern Ethiopia [19], limited attention has been given to young adults, who are at particularly high risk of SRH issues. Furthermore, no studies have explored barriers and facilitators for SRH uptake among AYAs in the Sidama region. Previous research has also not ranked identified service barriers and facilitators according to their importance to AYAs, making it challenging to prioritise interventions effectively. Therefore, this study aimed to explore barriers and facilitators of SRH service uptake among AYAs and rank the identified factors according to their relative importance.

## Methods

### Study design

We conducted a qualitative study using an inductive approach to allow themes and patterns related to preferences for SRH services to emerge from the data. Unlike deductive approaches, which are guided by predetermined frameworks, an inductive approach allows researchers to identify factors that may not have been anticipated, especially when examining sensitive topics such as SRH [23]. We conducted focus group discussions (FGDs) with AYAs and expert stakeholders (SRH, AYAs, health professionals) to comprehensively identify barriers and facilitators to SRH uptake. At the end of each FGD with AYAs, participants ranked the barriers and facilitators identified during discussion according to how strongly these factors would influence their decisions.

### Study setting and period

This study was conducted in the Central Sidama Zone, Sidama region in southern part of Ethiopia from October to December 2024. The Central Sidama Zone shares borders with all other zonal administrations in the Sidama region. Data collection took place in Yirgalem City, the administrative capital of the Central Sidama Zone. The city is situated approximately 326 kms south of Addis Ababa, the capital city of Ethiopia. In 2024, Yirgalem city had an estimated population of 90,730 (comprising 44,458 males and 46,272 females) [24]. As official age-disaggregated data were unavailable for Yirgalem City, the AYA population was approximated using Ethiopia’s national age distribution, estimating about 28,580 residents were aged 10–24 years. The city is administratively divided into 13 *kebeles*, the lowest administrative units in Ethiopia.

The Sidama region was purposively selected because it is one of Ethiopia’s newly established regional states and is undergoing important administrative and health system development. In this context, locally generated evidence is particularly valuable for informing regional health planning, resource allocation, and service improvement.

### Sample size and participant recruitment

Eight FGDs, each comprising 6–8 participants aged 15–24 years, were conducted, four with female participants and four with male participants. The discussions were held separately to encourage open and comfortable dialogue [25]. Data collection continued until data saturation was reached. In addition, a supplementary FGD were conducted with experts. Expert participants were invited from diverse professional backgrounds, including healthcare providers from Yirgalem Hospital (pharmacists and midwives), educators from local schools (teachers and heads of student counselling), government officials (heads of Dale District and Yirgalem City’s Female, Youth, and Social Affairs departments), and representatives from non-governmental organisations (Youth Development Officers). The expert FGD was conducted after the AYA FGD to validate the factors identified by AYAs and to gain further understanding from the experts' perspectives.

Participants were purposively selected and invited to take part in the FGDs. AYA participants were recruited from three distinct settings in Yirgalem City: Yirgalem High School, the local community, and the Talita Rise-up Orphanage Centre. These varied data recruitment sites were chosen to capture a broad range of perspectives. Participants were selected to reflect variation in age and educational status.

### Data collection tool and procedure

The FGD guide was prepared based on similar published studies [26] and consisted of open-ended questions with follow-up prompts. The guide covered broad health-related challenges of AYAs and SRH-related questions with a focus on SRH service accessibility, barriers to utilisation, equity and rights-based services, and adolescents' involvement. The participants were encouraged to discuss issues beyond the questions included in the FGD guide. The guide was prepared in English (*Supplementary file 1*) and translated into the national language, *Amharic*.

The FGD were conducted in *Amharic*, which is the national working language of Ethiopia and is commonly spoken in Yirgalem City. Amharic was used to ensure that participants from different linguistic backgrounds could express their views clearly. The moderators’ assistants were native Sidama (Sidaamu Afoo) speakers and were able to clarify language-related matters when required. However, no formal translation was needed during data collection.

FGDs were conducted separately for female and male AYAs to provide a comfortable and open environment for discussing sensitive SRH issues. For the female FGDs, a female moderator and the moderator assistant received one day of training. The female moderator held a master's degree, and the assistant held a bachelor's degree in health sciences. A female assistant moderator facilitated the FGDs with female AYA participants. The primary investigator and a male assistant moderator facilitated the male AYAs and expert FGDs.

Facilitators were trained by the first author and took detailed field notes throughout the discussions, including the key factors identified by participants. These notes were later used to guide participants in ranking the identified barriers and facilitators based on their perceived importance.

All FGDs, those conducted at Talita Rise-up Orphanage Centre, were held in Yirgalem High School. A quiet classroom was pre-arranged to avoid potential distractions and ensure confidentiality. The remaining two FGDs were conducted in a secure room at the Talita Rise-up Orphanage Centre. All FGDs, including with stakeholders, were audio-recorded. To maintain confidentiality and data security, recordings were transferred daily to the primary investigator’s password-protected computer and subsequently deleted from the recording device. The recordings were later transferred to Curtin University’s secured research drive, a shared network drive for storing research data.

The FGD moderator compiled a list of barriers and facilitators identified during the discussion, which was then used for the ranking exercise. Participants in each FGD were presented with the list of barriers and facilitators and ranked them according to their preferences.

### Data analysis

We conducted a thematic content analysis following Braun and Clarke’s six-phase approach [27]. First, all FGDs were transcribed and translated from Amharic into English to facilitate analysis. We familiarised ourselves with the data through repeated reading and listening to the recorded audio. In the coding phase, meaningful segments of text were systematically assigned initial codes. These codes were then organised and examined for patterns to generate potential themes. The provisional themes were reviewed against the dataset to ensure internal coherence and distinction. Finally, themes were refined, clearly defined, and named to guide the writing of the final report.

The transcription process was conducted by the assistant moderator, who was experienced in qualitative data collection and familiar with the content and context of the discussions. The principal investigator and first author (MBA) reviewed the transcribed data for consistency and completeness. The analysis was conducted using NVivo 15 [28].

The ranking results were analysed using STATA 18 MP [29]. A mean score was calculated for each attribute, and the attribute with the lowest mean score was considered the most important in influencing AYAs' decisions to use SRH services. If an attribute was not mentioned during a discussion and was not ranked, and was assigned the lowest rank [10] when calculating the mean score.

## Results

### Study participants’ characteristics

A total of 56 AYAs and seven SRH experts participated in the FGDs. Half of the AYA participants were male, and 57% of the expert participants were male. The mean age of the AYA participants was 18.2 years (standard deviation [SD]: 2.5), and 68% were adolescents aged 15 to 18 years. Most participants (96%) of participants were from urban areas, and 52 (93%) were students, and 50 (89%) relied on family support as their primary source of income (Table 1).

**Table 1:** Sociodemographic characteristics of FGD participants (AYAs and experts), Yirgalem, Sidama, Ethiopia

Variable	Category	Frequency (n)	Percentage (%)
*AYAs (n* = *56)*			
Gender	Male	28	50.0
	Female	28	50.0
Age group (years)	15–18	38	67.9
	19–24	18	32.1
Age group (years)	Age, mean (SD)	18.2 (2.5) *	
Marital status	Single	55	98.2
	Married	1	1.8
Residence	Urban	54	96.4
	Rural	2	3.6
Occupation	Student	52	92.9
	Other	4	7.1
Religion	Protestant	40	71.4
	Orthodox	10	17.9
	Other	6	10.7
Source of income	Family or partner support	50	89.3
	Own support from employment	6	10.7
Paternal education	No formal education	16	28.6%
	Primary (1–8)	27	48.2%
	Secondary (9–12)	7	12.5%
	Tertiary and above	4	7.1%
	Unknown	2	3.57%
Maternal education	No formal education	22	39.3%
	Primary (1–8)	30	53.6%
	Secondary (9–12)	2	3.6%
	Tertiary and above	2	3.6%
*SRH experts (n* = *7)*			
Gender	Male	4	57.1%
	Female	3	42.9%
Age group (years)	Mean (SD)	37.6 (9.9) *	
Religion	Orthodox	5	71.4%
	Protestant	2	28.6%
Experience in current position (years)	Mean (SD)	11 (12.6) *	
Total work experience (years)	Mean (SD)	15.3 (11.2) *	

*SD* Standard deviation, *AYAs* Adolescents and young adults, *SRH* Sexual and reproductive health

^*^Mean and standard deviation estimates

The mean age of expert participants was approximately 38 years (SD: 9.9). Regarding professional background, the participants represented various institutions, including hospitals, schools, government offices, and a nongovernmental organisation (NGO). Their mean total work experience was about 15 years (SD: 11.2) (Table 1).

### SRH barriers and facilitators

AYAs and experts identified a range of barriers and facilitators influencing young people's use of sexual and reproductive health (SRH) services. Through coding and analysis of the FGD, four main themes emerged: health system and policy environment, service access and delivery, user experience and outcomes, and sociocultural and economic factors.

## Health system and policy environment

### Health system infrastructure and resources

AYAs identified the cost of services, distance to health facilities, availability of medicines and medical supplies, and health provider characteristics as significant barriers and facilitators to accessing SRH services. Although some SRH services were officially provided free of charge, participants noted that the unavailability of medications and medical supplies at public facilities often forced them to purchase these items from private pharmacies. This additional out-of-pocket expense posed a substantial burden, as medications in private pharmacies were unaffordable for many young people. An expert participant described the issue as follows:

*“… another major issue is the cost of contraceptives. In the past, condoms and other birth control methods were provided for free, but now they come with a high price. This change has made accessing these services difficult for many people.” (Adolescent and young adult expert)*

Due to cost constraints, many AYAs reported a preference for public health services despite concerns about the quality of care they might receive. Although private health facilities were generally preferred because of their perceived higher quality services, cost was a significant barrier to access. Some participants mentioned that they might seek care from spiritual or religious places instead. A male participant described the situation as follows:

*“…money is a big problem for many people in rural areas. In these areas, there is often a shortage of money, which prevents people from seeking medical help. Instead, some people rely on spiritual or religious practices, like going to a Prophet's Church to testify about their illness, including HIV. They may avoid traditional treatments or health centres and publicly share their struggles as part of their faith.” (Male adolescent or young adult from Yirgalem High School)*

The availability of medicines and medical supplies, such as testing kits, syringes and needles, and gloves, was identified as an important factor influencing SRH service uptake among AYAs. AYAs perceived hospitals and private clinics as having better availability of medication and medical supplies than public health centres. They also perceived these facilities as having well-trained health providers.

*“Most young people prefer to be treated at a hospital if they have a sexually transmitted disease (STD). … because they think that there is enough medicine in the hospital and that they will find more and better [qualified] doctors, they prefer to be treated in the hospital.” (Adolescent and young adult expert)*

*“Sometimes, the preferred birth control method may not be available at the health facility. For example, the contraceptive inserted in arm [implant] may not be available in the health centre.” (Female adolescent or young adult from Yirgalem High School)*

AYAs expressed mixed views about the distance to health facilities when seeking SRH services. While some preferred to access services near home for convenience, others preferred facilities farther away due to privacy concerns. Many AYAs were worried about being seen by someone they knew or feared that healthcare providers might disclose their visit to family members.

*“Distance is a big challenge when trying to access service. It may prevent her [female AYA] from seeking treatment if the health facility is too far away. She [female AYA] may also have to spend money on transport, making it harder for her to access the care she needs.” (Female adolescent or young adult from Yirgalem City)*

*“… I would not go to the nearby health facility. Instead, I would choose a hospital that is far away. The main reason for this is to protect my secret [privacy]. If I go to Yirgalem Hospital, I might be recognised because many people in the area know my family, and the nurses are also from here [same residence]. They might ask questions like, “Why are you here?” or gossip about my visit. To avoid this, I would prefer to go to a more distant hospital where I can get treatment without the risk of being recognised.” (Male adolescent or young adult from Yirgalem High School)*

AYAs also noted preferences regarding specific health provider characteristics such as age and sex. However, these preferences varied. Some AYAs reported feeling more comfortable with older providers of the opposite sex, perceiving them as more experienced and less judgemental. In contrast, others expressed a strong preference for younger providers of the same sex, citing shared experiences and reduced embarrassment when discussing sensitive SRH issues.

*“I prefer a health provider to be a woman because, as another woman, she understands women’s issues better than men and can easily help me. If it [health provider] were a man, I wouldn’t feel as comfortable talking freely and might hide things. That is why I would prefer a female professional.” (Female adolescent or young adult from Yirgalem City)*

*“The age and sex of health providers affect service use. One time, a female doctor asked me to lower my pants [for physical examination], and I was very uncomfortable.” (Male adolescent or young adult from Yirgalem High School)*

### Policies and procedures

Both AYAs and expert participants highlighted the influence of legal and policy issues on young people's use of SRH services. For example, participants mentioned that some AYAs underwent unsafe abortions because they believed abortion was illegal in Ethiopia unless certain conditions were met. Expert participants also noted that health facilities sometimes reported inaccurate information about their services, hindering appropriate planning. In addition, participants expressed concern that the government did not give sufficient attention to important SRH issues such as HIV/AIDS.

*“There is no supportive environment for young people, despite three out of four of the population being young. They are prone to illegal activities and protests. There are no youth-friendly sites in the community. You cannot believe it — there are 345 Khat chewing houses [social or business spaces where people gather to chew the Khat leaves, a green leafy stimulant plant commonly used in some East African communities] in Yirgalem city. However, there are very few youth-friendly sites in the city. Is it surprising that these people [AYAs] are ending up like this [addicted]? No, it is not surprising! How many young people are in these houses [chat selling houses]?’’ (Adolescent and young adult expert)*

Legal provisions intended to protect AYAs are often poorly enforced, limiting their practical application. An expert working with AYAs highlighted the gap between the existence of protective laws and their implementation on the ground.

“*Because society and legal bodies remain silent, many young people, especially children, are being exposed to various addictions at an early age. For example, although the law prohibits the sale of beer to those under 18 and betting houses claim they do not allow anyone under 18 to participate, children still engage in these activities*.” *(Adolescent and young adult expert)*

## Service access and delivery

### Awareness and information provision

Participants stated that AYAs, particularly those from rural areas, often faced substantial gaps in awareness and information regarding SRH. They discussed how many AYAs were unfamiliar with the symptoms of STIs, leading to delays in seeking care.

*“What I'm talking about now is the situation where sexually transmitted disease (STD) occurs among young people in rural areas. Most young people are unaware of the symptoms, so they remain at home [not seeking treatment]. However, when they start experiencing itching in the genital area, unusual discharge with a smell, and the symptoms worsen, they eventually seek help at a medical institution.” (Adolescent and young adult expert).*

Misinformation and rumours, such as beliefs that contraceptives cause infertility, also discouraged service use, especially among adolescent girls. Additionally, limited understanding of safe abortion options contributed to unsafe practices, with fear of pregnancy often outweighing concerns about STIs such as HIV.

*“Rumours about contraceptive services are another barrier to using them. For example, some people believe that birth control can cause infertility. If someone told me that the contraceptives could cause infertility, she would decide not to use the service rather than asking health professionals.” (Female adolescent or young adult from Yirgalem High School)*

*“Of course, there are plenty of illegal abortion practices in the community. In fact, nowadays, many young people fear unplanned pregnancy more than HIV. If you ask every pharmacy, about 90% of their sales could be postponed.” (Adolescent and young adult expert)*

AYAs accessed SRH information through multiple sources, including peers, online platforms, healthcare providers, and by observation of older individuals. However, unfiltered exposure to sexual content, especially through smartphones, may influence risky behaviours, such as unprotected sex, potentially leading to serious health consequences.

*“Many students have touchscreens [smartphones], through which they access various content, including sexual content. I am speaking the truth—this exposure encourages them to experiment with inappropriate behaviours, which can result in serious health problems. They engage in unprotected sexual intercourse, which can lead to conditions such as fistula.” (Adolescent and young adult expert)*

### Service delivery

Participants highlighted that service delivery factors including privacy, cleanliness, inconvenient opening hours, long waiting times, limited choice of services, and health providers' attitudes and communication styles played a substantial role in shaping AYAs' experiences.

Privacy was the most frequently discussed issue, with many AYAs expressing concerns about potential breaches of privacy when seeking SRH services at public health facilities. In some instances, participants were also concerned that healthcare providers might disclose their visits to their parents, further compromising confidentiality.

*“The main reason people avoid government health facilities is fear that their family, neighbours, or church will find out about the pregnancy or abortion. To keep it a secret, many choose cultural treatments because they are done privately, even though it is illegal. Some also try to end the pregnancy themselves by using traditional remedies or buying medicines from pharmacies. They do all this to ensure their community or family doesn’t find out.” (Adolescent and young adult expert)*

Participants expressed mixed views about the role of health providers in maintaining privacy. Some AYAs believe that healthcare providers were professionals who would keep their information private. Others were concerned that providers might share their personal information with others.

*“From my experience, health professionals keep things private. When my friend got pregnant, they spoke to her in a separate room. I believe health providers keep our secrets.” (Female adolescent or young adult from Yirgalem City)*

*“The health provider maintains confidentiality if we do not know them personally. However, they often fail to keep matters confidential if we do know them.” (Female adolescent or young adult from Yirgalem City)*

Participants were concerned about the limited availability of preferred SRH service options, especially contraceptive methods. They explained that providers sometimes did not offer the specific contraceptive method they sought, or the facility did not have their preferred method in stock. In some cases, they also felt that health providers did not respect their preference regarding contraceptive methods. This could discourage them from seeking care. One participant described this concern as follows:

*“The other problem is the lack of choice in services. For example, my friend [female friend] and I went to the health centre because my friend wanted to get injectable birth control. However, they [health providers] insisted on giving what they preferred. … We [her friend] specifically requested a three-month injectable contraceptive, but they [health providers] recommended the loop contraceptive instead. There was limited communication for why they do not give the injectables” (Female adolescent or young adult from Yirgalem High School)*

Cleanliness, opening hours, and waiting times were identified as key factors influencing SRH services uptake. Public health facilities were often perceived as less clean and associated with longer waiting times than to private facilities. Many participants expressed a preference for accessing SRH services outside school hours.

*“The cleaning situation is very difficult. You are sick, and you risk getting another disease. You will find the smell very bad when you go to the hospital. If you want to use the toilet, it is unimaginable. There are several patients in one room. Patients there are adding another disease, and many of them are sleeping in the same room. It is very worrying because her room is small, and it smells.” (Male adolescent or young adult from Yirgalem High School)*

*“I finish school after 3 pm, and the professionals have already left when I go to the health centre. Because of this, I either leave school early or miss something else, but if I go to the health facility at my preferred time, I often don't receive treatment.” (Male adolescent or young adult from Talita non-governmental organisation)*

*“I recently visited a health centre and waited three hours but received no service. Despite the large number of patients in the waiting area, the health professionals walked around, saying, "I feel hungry." This shows a lack of responsibility and respect for the people they are supposed to serve.” (Male adolescent or young adult from Yirgalem High School)*

Health provider attitudes and communication significantly influenced AYAs choice of health services. One female AYA from Yirgalem High School noted that respectful treatment was grounded in medical ethics, although not all providers upheld these standards.

*“Most health professionals, but not all, will treat us with kindness and respect. This is because medical ethics require them to treat patients well, and doctors have promised to help the community with honesty and humility. Those who follow these principles will offer a warm and supportive environment. However, some may not act according to these ethical standards. It is not fair to say every professional is the same.” (Female adolescent or young adult from Yirgalem High School)*

## User experience and outcomes

### Previous experience

Previous healthcare experiences, both positive and negative, shaped AYAs decisions to seek sexual and reproductive health (SRH) services. Encounters with the broader health system, even when unrelated to SRH care, influenced perceptions of service quality and trust. Negative experiences, such as the unavailability of essential supplies, inadequate treatment, or misdiagnosis, may undermine confidence in public facilities and deter future use.

*“The health centre lacks essential medicines and medical supplies. My family and I once went to a health centre but could not find a blood pressure-measuring instrument. Because of this experience, I do not want to receive care at a facility that does not have adequate medical supplies.” (Male adolescent or young adult from Yirgalem City)*

Such experiences contributed to mistrust and may prompt AYAs to seek SRH care elsewhere, often in private facilities perceived to offer a better quality of care.

*“If I had SRH problems, I would go to a private clinic [do not want to go to the health centre]. For example, one of our friends fell and started drooling. We took him to the health centre, where they treated his wound and sent him home without proper care. When he later went to the hospital, they referred him to Hawassa [Sidama Capital City], suggesting it might be a seizure since he had never experienced it before.” (Female adolescent or young adult from Yirgalem High School)*

### Perceived SRH outcomes

The perceived outcomes of SRH services, including treatment side effects and effectiveness, were important, influencing service uptake. For example, participants perceived some contraceptive methods as potentially causing infertility.

*“I do not want to use the service [contraceptive methods] because there are other natural ways to prevent pregnancy, and these [contractive methods] have side effects. For example, there is a calendar method [rhythm method]to prevent pregnancy.” (Female adolescent or young adult from Yirgalem High School)*

## Sociocultural and economic factors

Sociocultural and economic barriers significantly influenced SRH services uptake among AYAs. Factors such as peer pressure, religious beliefs, and stigma discouraged AYAs from seeking SRH services or were associated with shame. In addition, economic hardships, including poverty, unemployment, and urban migration, limited young people's access to affordable and youth-friendly healthcare options.

Peer pressure was perceived as an important factor influencing AYAs involvement in risky sexual behaviours. AYAs may follow their peers to fit in with their group, potentially placing their sexual health at risk.

*“There’s also pressure from friends to do what they’re doing or to fit in. It’s easy to start watching videos online for pleasure, and over time, this can lead to having sex with different women. While it might seem exciting at the time, it can also bring challenges and consequences you don’t always think about.” (Male adolescent or young adult from Talita non-governmental organisation)*

In addition to peer pressure, limited family support may increase AYAs' exposure to risky activities. AYAs mentioned poor communication between their parents. One participant described his relationship with his father as follows:

*“I am sure my father will kill me if I tell him I have sexual and reproductive health problems, or my family will kick me out of the house. I am scared [of their families], but at least I will talk to my friends.” (Male adolescent or young adult from Yirgalem High School)*

Religious norms and beliefs play a role in shaping attitudes toward SRH service utilisation. In some communities, religious teachings discourage the use of contraception and portray premarital sexual activity as morally unacceptable. These perspectives can create fear, guilt, or shame in young people.

*“The influence of religion can also be a barrier. For example, some religious teachers say that using contraception is like destroying a child created by God.” (Female adolescent or young adult from Yirgalem High School)*

Fear of stigma and social exclusion remained a critical barrier to accessing SRH services among AYAs, especially among unmarried mothers. Social norms that condemn premarital sexual activity, pregnancy outside marriage, or living with conditions such as HIV often led young people to avoid seeking care, even when medical attention was urgently needed.

*“In society, if a woman gives birth to a child or becomes pregnant outside of marriage, it is considered shameful and brings great disgrace to her parents. Fearing stigma, she often avoids seeking care at government medical institutions.” (Adolescent and young adult expert)*

*“For example, individuals diagnosed with HIV or other diseases may face social exclusion. People often hesitate to associate with someone living with HIV, leading to stigma and discrimination within the community. This social isolation can significantly impact those affected emotionally and psychologically.” (Male adolescent or young adult from Talita non-governmental organisation)*

Poverty and low socioeconomic status were major drivers of vulnerability among AYAs, particularly in relation to SRH risks. Economic hardship may push AYAs, especially young women, into situations such as transactional sex or early sexual engagement as a means of financial survival. The pressure to support themselves or their families may lead to difficult choices, often made without safer alternatives.

*“I think it’s often women who are pushed into situations like this [prostitution], especially when facing economic struggles. No matter how difficult things are at home, I don’t believe boys are as likely to go down that path. Boys might feel less pressure to change their family’s financial situation in such ways. Conversely, women might feel they have no choice and see it as an easy way to make money. For boys, peer pressure at school or among friends is more likely to push them into similar situations. Many young people, especially adolescents, are influenced by their peers and exposed to online adult content, which can lead them into these kinds of experiences.” (Male adolescent or young adult from Talita non-governmental organisation)*

Urban migration was perceived as a major factor increasing the vulnerability of AYAs, particularly young women, in the Sidama region. Many children and AYAs were sent to cities such as Yirgalem by their families because of economic hardship, often to work as housekeepers or seek better opportunities. However, in the absence of adequate protection and support, these young migrants may end up working in bars and engaging in sexual activities with multiple partners to earn an income. This exposes them to a range of health risks, including unwanted pregnancies and sexually transmitted infections.

*“There is a high migration rate of young people to the city [Yirgalem]. Often, these young people turn to illegal brokers who involve them in sexual activities and even smuggle them to other places. If someone experiences sexual assault and wishes to pursue legal action, the legal system is very time-consuming. There are language barriers and a lack of knowledge, which further complicate matters. As a result, unsafe and illegal abortions have destroyed the lives of many young girls.” (Adolescent and young adult expert)*

## Ranking exercise

At the end of each FGD, participants ranked the barriers and facilitators they believed influenced their decisions to use SRH services. Each group identified between 7 and 10 relevant barriers and facilitators, which they then ranked according to perceived importance. In total, 12 unique barriers and facilitators were identified across all FGDs. Of these, privacy, cost, availability of medication, quality of services, waiting time, and provider attitude were consistently cited by all groups. In contrast, provider characteristics (such as age and sex), incentives, and side effects were mentioned in only one FGD. Based on the mean score rank, quality of service, privacy, and cost were the top three high ranked barriers and facilitators influencing SRH service uptake among AYAs (Table 2).

**Table 2:** Ranking of barriers and facilitators identified during FGDs

Attributes	Barriers	Facilitators	No of FGDs nominated for the attribute	Mean rank score*	Rank
Quality	Low quality	High quality	8	2.7	1
Privacy	Not private	Private	8	2.9	2
Cost	High cost	Free/low cost	8	3.8	3
Availability of medication	Limited availability of medication	Adequate availability of medication	8	3.8	3
Provider attitudes	Judgmental/disrespectful provider attitude	Friendly, respectful, and non-judgmental provider attitude	8	5.4	5
Waiting time	Long waiting time	Short waiting time	8	5.9	6
Cleanliness of the health facility	Unclean health facility	Neat and tidy health facility	7	6.6	7
Distance to health facility	Facility located far away	Nearby health facility	6	7.4	8
Opening hours	Limited or inconvenient opening hours	Flexible working hours including weekend and after hours	3	9	9
Provider characteristics	Provider characteristics such as age and sex	Provider characteristics such as age and sex	1	9.4	10
Incentives	No incentives provided	Incentives provided	1	9.4	10
Side effects	High treatment side effects	No/minimal side effects	1	9.6	12

^*^If an attribute was not ranked by a participant, it was assigned the lowest rank (10^th^ rank) when calculating the mean rank score; FGD: focus group discussion; No: number

## Discussion

This study explored barriers and facilitators for SRH services use among AYAs in the Sidama region, Ethiopia. The findings were organised into four overarching themes: health system, policy and procedural gaps, service delivery, access to information, user experience and outcomes and sociocultural and economic factors.

Health system infrastructure and resources influenced AYAs' preferences for accessing SRH services. Participants highlighted limited availability of medical supplies, inadequate staffing, and poorly maintained facilities as major deterrents to seeking care. Infrastructure is one of the component of healthcare quality [30]. Participants were also concerned about the cost of services, particularly when services were unavailable in the public health facilities. As most students have no stable source of income, they could not afford to pay for health services. These findings align with previous research indicating that structural deficiencies in low-resource settings can negatively affect AYAs' engagement with SRH services [31–34]. Thus, health facilities should improve the availability of medication and medical supplies to reduce the financial burden on AYAs and improve SRH service uptake.

Various legal and procedural constraints influenced the uptake of SRH services. In 2005 Ethiopia liberalised its abortion law under certain circumstances. However, these circumstances may still hinder AYAs from accessing safe abortion services in health facilities [35]. Some participants reported seeking alternative options, as they were unable to access abortion services at health facilities unless they met the legal criteria for safe abortion. These alternatives were often unsafe and may have endangered their lives. Furthermore, there are challenges to enforcing existing laws that could lower the risk of SRH services. For example, even if the legal age of marriage in Ethiopia is 18 years, enforcement is limited. A qualitative study conducted in the Amhara region revealed that children were exposed to alcohol before reaching 18 years of age [36]. Government attention to the health sector also remains limited, as reflected in the declining trend in health expenditure as a percentage of Gross Domestic Product [37]. This highlights the need for more youth-responsive legal frameworks and stronger enforcement of existing laws to improve access to safe SRH services. Increased government investment in the health sector is also essential to address structural barriers that place AYAs at risk.

Service delivery factors and experiences, including cleanliness of the environment, privacy, and waiting time, were a highly ranked factor influencing SRH service utilisation. The perceived lack of privacy, cleanliness, and youth-friendly spaces particularly discouraged young people from visiting public health institutions, a barrier also highlighted in studies from other sub-Saharan African contexts. [31, 33, 38]. AYAs' previous experiences at health facilities influenced their future use of SRH services, even when those visits were unrelated to SRH. This highlights the need for respectful, youth-friendly care across all services to build trust and encourage future health-seeking behaviour. Strengthening system-level collaboration is essential to ensure coordination across all points of care.

Limited awareness and information gaps were key challenges to SRH services uptake among AYAs, particularly those residing in rural areas. Furthermore, misinformation about SRH services, especially contraceptives, was common. For example, some participants believed that contraceptive use could result in infertility. This finding was consistent with a recent study examining contraceptive perception among rural communities in Ethiopia [39]. This underscores the need for community-based education and communication strategies to address misconceptions and improve SRH knowledge among rural AYAs.

Perceived SRH outcomes, including anticipated side effects, perceived infertility, and social judgement, also influenced service uptake. These perceptions, based on previous experience or misinformation, may deter AYAs from using services. Previous studies have similarly shown that perceived effectiveness and side effects influence AYAs' preferences SRH services uptake [40–42]. This highlights the need for culturally sensitive communication and counselling to address fears and build confidence in SRH services among AYAs.

### Strengths and limitations

This study has several strengths that contribute to the depth and credibility of its findings. First, it draws on diverse perspectives by including AYAs from schools, community settings, and NGO, which enhances relevance of the data across different segments of the AYA population. Second, the inclusion of expert participants, such as healthcare providers, government officials, and youth workers allowed for a broader understanding of the structural and systemic factors influencing SRH service access. Third, the study incorporated a ranking exercise of service barriers and facilitators, which provided crucial information on the relative importance that AYAs place on different aspects of SRH services.

The following limitations should be considered when interpreting these findings. The study was limited to Yirgalem City; therefore, the perspectives of AYAs living in rural and remote areas may not have been fully captured. In addition, social desirability bias may have influenced participants’ responses, particularly on sensitive topics such as sexuality, abortion, and substance use. Although efforts were made to ensure confidentiality, the group setting of FGDs may have limited some participants’ willingness to express their views openly.

### Implications and recommendations

This study has important implications for improving AYAs' access to SRH services in the Sidama region. The findings emphasise that service quality, privacy, and cost are the most influential factors shaping SRH service uptake, highlighting the need for focused interventions addressing these concerns. Efforts to improve SRH access should go beyond service availability and include health system reforms that ensure privacy, reduce financial barriers, and enhance service quality. Furthermore, the identified barriers and facilitators provide a valuable foundation for future research, such as stated preference studies, to inform youth-centred and evidence-based SRH service planning.

## Conclusions

This study identified the factors influencing AYAs' utilisation of SRH services in the Sidama region of Ethiopia. SRH service use was shaped by interconnected factors, including health system infrastructure, policy gaps, service delivery, access to knowledge and information, previous experiences and perceived outcomes, and sociocultural and economic constraints. Service quality, privacy, and cost were the most frequently prioritised issues across participant groups. These findings informed the identification of key attributes that may be useful for future research quantifying AYAs’ preferences for SRH services. Policymakers are encouraged to prioritise interventions that improve the factors most highly ranked factors by participants.

## Supplementary Information

Supplementary Material 1.

## Data Availability

No datasets were generated or analysed during the current study.
