# Supplementary material for: Barriers and facilitators to sexual and reproductive health services among adolescents and young adults in Sidama region, Ethiopia: a qualitative study
Source: Reprod Health. 2026 Jun 24;23:167. doi: 10.1186/s12978-026-02400-2 (PMC13551953; doi:10.1186/s12978-026-02400-2)
Supplement: Supplementary file 1 [file 12978_2026_2400_MOESM1_ESM.docx]

### FGD Guide Questions

**General questions**

1. What are the general health problems among young people in this community?

2. What reproductive health (sexuality-related issues) challenges are common among young people? Probe for STIs, including HIV, if not mentioned.

3. What do you think are the causes of these sexuality-related health problems confronting young people? Please ask this question for each problem identified. Prob with (home, school, peer or online)?

**Accessibility of services**

4. What do young people usually do if they think that they have a sexually transmitted disease? Where would they go to get treatment? Are there any places that people like better or worse than others? What are the reasons for these preferences?

5. Where can you get or ask for family planning and reproductive health information and services in your area? Are they free? If not, how much do they cost? Would you use contraceptives if they were free from the health centre? Why or why not?

6. What do you know about the sexual and reproductive health (SRH) services rendered in the public health facilities in your community? Probe: What are their working days and hours? In what ways are these services welcoming or not welcoming?

7. What other strategies can we use to ensure that family planning and reproductive health information and services are accessible to those who need them most?

**Service acceptability**

8. What are your perceptions about the environment, setting, and procedures of health facilities offering SRH for adolescents and young people in your community? Probe for cleanliness; probe for adequacy of opening hours

**Barriers to utilisation of services**

9. What are the barriers that adolescents and young people experience in utilising sexual and reproductive health services in primary health facilities in the Sidama region? Probe for cost, distance, health worker attitude, waiting time, and caregiver (parent/guardian) support.

a. What do you think hinders people from using contraceptives?

b. What hinders you from accessing family planning contraceptives from the health centre?

**Privacy and confidentiality:**

10. What is your perception of service providers’ sensitivity to the needs of young people, their privacy and confidentiality in sexual and reproductive health (SRH) service provision in public health facilities in your community?

**Equitable & rights-based services**

11. What rights do you have in accessing sexual and reproductive health (SRH) services in public health facilities within your community?

12. What are your perceptions about how health workers treat adolescents and young people seeking sexual and reproductive health (SRH) services in public health facilities in your community?

**Adolescents' and young adults' involvement**

13. In what way are young people involved or engaged in the planning, implementation and evaluation of sexual and reproductive health (SRH) for adolescents and young people within the Sidama region?

14. What mechanisms are available to involve young people in providing health services, including serving as peer educators and community resource persons?

15. What mechanisms are available to involve young people in monitoring health services, including functioning as members of relevant management committees?

**Facilitators of utilisation of services**

16. What factors promote adolescents and young people utilising sexual and reproductive health services in primary and secondary health facilities in Sidama State?

17. What should the health facility do to improve your experience in accessing family planning information and reproductive health services?

**Conclusion**

18. How has a national emergency (conflict and COVID) impacted your ability to assess sexual and reproductive health (SRH) at a public health facility?

19. What other information may be useful for this study?

**Priority list**

20. How do you prefer SRH services to be provided for people in your age group in the public health facilities in your community?

21. You have been given a sheet of paper each. Please list five ways that you prefer for sexual and reproductive health care to be offered to you as a young person and rank them in order of importance.

### Ranking survey format

Please rank the listed attributes from most important to least important for influencing the decision to seek SRH service in the health facility.

| Attribute list | Rank | Remark |
| --- | --- | --- |
| 1. |  |  |
| 2. |  |  |
| 3. |  |  |
| 4. |  |  |

### Expert panel discussion guide

1. What are the sexual and reproductive health challenges of adolescents and young people that you are aware of?

2. Adolescents and young adults often have health needs that relate to their sexual and reproductive health, such as information about ways of preventing pregnancy or sexually transmitted infections, including HIV. Where do young people go to receive this information or treatment when they need it?

3. What options do adolescents and young adults use rather than going to a government hospital or health centre? Probe for private health facilities.

4. What are the characteristics of good health facilities for addressing the sexual and reproductive health challenges of adolescents and young adults from your perspective as an adolescent/young person?

5. Discuss the summary of focus group discussions and set attribute levels for the identified attributes.

a. Please comment on whether you think it is important to them and why you responded.

b. What do you think are the characteristics that are important for young people?

c. Please mention any characteristics that are not included in the FGD result.
